# How to Measure Human-Dog Interaction in Dog Assisted Interventions? A Scoping Review

**DOI:** 10.3390/ani14030410

**Published:** 2024-01-26

**Authors:** Marta De Santis, Lorena Filugelli, Alberto Mair, Simona Normando, Franco Mutinelli, Laura Contalbrigo

**Affiliations:** 1 National Reference Centre for Animal Assisted Interventions, Istituto Zooprofilattico Sperimentale delle Venezie, Viale dell’Università 10, 35020 Legnaro, Italy; mdesantis@izsvenezie.it (M.D.S.); lfilugelli@izsvenezie.it (L.F.); fmutinelli@izsvenezie.it (F.M.); lcontalbrigo@izsvenezie.it (L.C.); 2 Department of Comparative Biomedicine and Food Science, Università degli Studi di Padova, Viale dell’Università, 14, 35020 Legnaro, Italy; simona.normando@unipd.it

**Keywords:** animal assisted intervention, therapy dog, dog-human interaction, dog-handler, dog-human relationship, dog-human bond

## Abstract

**Simple Summary:**

A particular type of human-dog interaction is established in dog-assisted interventions (DAIs). This interaction is based on strong human-animal cooperation and the possibility of mutual benefit during the intervention and involves complex dynamics and multisensory channel communication. The issue of measuring human-dog interaction is not new to the field of anthrozoology, but it becomes even more exciting in DAIs, given the central role that the human-animal relationship plays. In this scoping review, we look at the methods and tools that have been used to date to analyse dog-human interaction, relationships and bonding in this context. The results highlight the need for further development and refinement of the tools in terms of validity and reliability. Other emerging trends in research are the need to take into account the perspective of the dog involved and the influence of the dog-handler relationship on the outcomes of interventions in terms of the well-being and functionality of the dyad. The identified methods and tools can be used by both researchers and practitioners to further investigate aspects of human-dog interaction in the field of DAIs.

**Abstract:**

Human-dog interaction is the working tool through which the therapeutic, educational and recreational goals of dog-assisted interventions (DAIs) are achieved. A better understanding of the characteristics of this interaction could improve the effectiveness of DAIs. This scoping review addresses the question: how has the human-dog connection been measured in the context of DAIs? After searching the Web of Science and Scopus platforms, only peer-reviewed, primary research studies reporting measures of therapy dog-human interaction, relationship and bond were included. A total of 70 included articles provided information on what was measured (interaction, relationship or bond) and how, as well as the general context (DAIs or experimental situations with therapy dogs). While the majority of the articles identified use behavioural analysis methods to analyse the interaction between the participant/recipient and the therapy dog during DAIs, it was possible to identify some more structured tools that assess the participant/recipient’s interaction, relationship or bond with the therapy dog, as well as tools that consider the animal’s perspective or focus on the dog-handler dyad, indicating growing areas of research. The tools and methods identified can be used by both practitioners and researchers to further explore aspects of human-dog interaction in the field of DAIs.

## 1. Introduction

In animal-assisted interventions (AAIs), human-animal teams are incorporated into formal human services in order to reach therapeutic, educational and recreational goals [1,2]. Although the working mechanisms of AAIs are not yet totally understood and need further study [3], the relationship and interaction with the animal appear to be key to their effectiveness and, when properly established and guided, can positively affect human health [4,5,6]. 

Over the past decades, a number of underlying mechanisms and theories have been called upon to explain how this interspecific relationship can be beneficial, for example, through social and emotional support or through the establishment of an attachment bond [3,5,7]. Beyond possible causes and concurrences, the human-animal relationship assumes a central role in AAIs, particularly in the light of the concept of One Health and One Welfare, which see the interconnectedness of humans, animals and their environment [1,5,6,8,9], aiming to create synergies for all the parties involved. As commented by Colonius and Earley [10], after all, it is an artificial compartmentalisation to separate human, social and animal welfare. In reality, they are interdependent. Ideally, therefore, an effective AAI will benefit the patient/user, be enjoyable for the animal involved and also facilitate the development of beneficial relationships in the context or environment in which it takes place. For this reason, in addition to asking “why” this interspecific relationship works, it is interesting to analyse “how” it works. 

In essence, the human-animal relationship itself can become the object of study, as it represents the actual working tool in AAIs [6]. The animal involved interacts with the patient/user but first establishes a special bond with the handler, requiring a high level of cooperation and interspecific communication to achieve the intervention goals. As described by Menna and colleagues [5], AAIs are “a system within which there are relational dynamics of living beings belonging to two different species.” A series of relational feedbacks take place during the AAIs, resulting in a mutual and reciprocal influence of the subjects and the establishment of synergistic interactions [4,5].

However, deepening knowledge about the characteristics that make this relationship mutually positive, effective and beneficial—and then somehow being able to assess it—is far from simple. As already pointed out by other authors [1,5], new approaches, instruments and research designs are needed to investigate what happens between humans and animals during AAIs, their relationship, and the way they influence each other. 

AAIs involve a variety of animal species, each with their own unique characteristics. Dogs are one of the species most frequently involved in AAIs [4,11]. Additionally, the long co-evolutionary history between humans and dogs has been the subject of numerous studies, revealing important facets of interspecific communication and bonding [4,12,13,14]. Therefore, the connection between dogs and humans in AAIs can be considered a good paradigm to explore. This scoping review focuses on the human-dog relationship in the context of dog-assisted interventions (DAIs) and addresses the question: how has the human-dog relationship in DAIs been measured so far? To answer this question, it analyses the scientific literature in order to identify the methods and tools that have been used to quantitatively assess the interactions, relationships, and bonds that take place between the dog and the human counterpart in DAIs. 

Preliminary searches through Google Scholar and Web of Science (WoS) were conducted, and no systematic or scoping reviews on the topic of measuring human-dog interaction in the context of DAIs were identified. However, two reviews by Wilson and Netting [15] and Samet et al. [16] examined the topic of human-dog interaction in general, providing an overview of the status of instrument development in the field of human-animal interactions (HAI), including some tools designed to evaluate therapeutic interventions. In particular, Samet and colleagues [16] highlighted the lack of measures for HAI in the field of assistance and therapy animals. Another article by Rodriguez and colleagues [17] discusses the state of assessment in HAI research, distinguishing between questionnaires, physiological measures and behavioural observations as categories of assessment. There are also reviews that analyse the literature on attachment and bonding in the dog-human dyad [7], or the determinants of a satisfying dog-owner relationship [18], or more generally, the scientific literature on human-animal interactions, relationships and bonds [19]. Although these sources are not focused on dog-human measurements in the context of DAIs, they will allow comparison and provide theoretical references, along with the abovementioned papers [1,3,5]. Furthermore, the initial literature search allowed us to identify the most commonly used terms to refer to the topic of this review: following Hosey and Melfi’s approach [19], we focused on the terms interaction, relationship, and bond. This review does not aim to delve into definitions of these terms, as their meanings sometimes overlap and are not consistently used across different disciplines. However, as Hosey and Melfi did [19], we use Hinde’s framework [19,20] to distinguish between the terms “interaction” and “relationship”. According to this framework, an interaction is “a sequence in which individual A shows behaviour X to individual B, or A shows X to B and B responds with Y”, while a relationship involves a series of interactions in time between individuals known to each other [20]. The term “bond”, which is more commonly used in companion animal and laboratory animal literature [19], refers to a “mutually beneficial relationship between people and animals that is influenced by behaviours considered essential to the health and well-being of both” [21]. Therefore, it appears that emotional and psychological components are added to the previously described terms of ‘interaction’ and ‘relationship’, implying mutual benefits for the individuals involved (for further information on this topic, refer to [19]). 

The objective of this scoping review is to assess the extent of the literature reporting methodologies and instruments used to assess the dog-human interaction, relationship and bond in the context of DAIs. The ultimate goal is to map out reliable tests and/or assessment tools that can be used (or developed further) not only in research but also during the interventions in order to provide support to DAI practitioners.

## 2. Materials and Methods

### 2.1. Protocol and Eligibility Criteria

A protocol for this scoping review has been drafted based on the Joanna Briggs Institute (JBI) methodology for scoping reviews [22] and the Preferred Reporting Items for Systematic Reviews and Meta-Analyses extension for Scoping Reviews (PRISMA-ScR) [23] and is available on request from the authors. 

Only published, peer-reviewed, primary studies with the following inclusion criteria were considered: the focus of the review is on the tools and methods used to measure the interaction, relationship, and bond between dogs involved in DAIs and humans (handlers and recipients). The participants in this scoping review are therapy dogs and their human counterparts (specifically, the handler and the patient/recipient of the intervention). Only dogs trained for DAIs or at least habitually involved in DAIs were considered for inclusion. Shelter dogs or family dogs (with no specified previous experience) involved in therapeutic, educational, or recreational intervention, as well as assistance dogs, were not included in this review. The reason for this choice is that therapy dogs involved in DAIs and assistance dogs have different training and lifestyles: a therapy animal is guided by the handler but works for the benefit of others, whereas assistance animals usually live with the person they assist, who becomes their handler [24]. Similarly, shelter dogs and family dogs with no experience with AAIs may not have developed a collaborative relationship with the handler, which is one of the fundamental elements of DAIs. In terms of measures and tools, the aim was to identify those that measure the interaction, relationship or bond with the therapy animal involved rather than with animals in general. In addition, tools designed to assess other constructs (e.g., dog personality or attitudes towards animals) were not included, nor were tools that did not specifically distinguish interaction with the dog from other interactions with the environment or other people.

For the purposes of the scoping review, the geographical context was left open (i.e., not limited to specific areas or countries), as well as the settings in which measurements are collected (e.g., natural environment, clinics or hospitals, farms, research settings, etc.). Given that DAIs are defined differently in different countries and cultures, the context of DAIs was deliberately not strictly defined, with the exception of the rules described about participants. Experimental settings with therapy dogs were also considered, with the aim of exploring the relationship, bond, and interaction between dogs and humans. No restriction was placed on quantitative study designs: experimental and quasi-experimental, as well as analytical and descriptive observational studies, were included. In order to drive the focus towards straightforwardly quantifiable measures of interaction, relationship, and bond, studies with a qualitative design, such as those based on focus groups, interviews and thematic analysis, although interesting, were excluded (e.g., [25]). Moreover, reviews, books, commentaries, editorials, letters and conference proceedings were excluded. Finally, due to time and resource constraints, only studies published in English were included, with no restriction on search dates.

### 2.2. Information Sources, Search, and Selection

To refine the search strategy and identify the most appropriate keywords, an initial limited search of Google Scholar, Scopus and the WoS Core Collection was undertaken. The Web of Science (WoS) and Scopus platforms were searched in July 2023. The WoS platform search included the following databases: WoS Core Collection, MEDLINE^®^ and SciELO Citation Index A. The search query is shown in Table 1. After checking for duplicates, two reviewers (M.D.S. and L.F.) performed the first step of the study selection process (title/abstract screening) and discussed any doubts to refine the screening rules. Full-text screening was then carried out independently by two reviewers (M.D.S. and L.F.). Any disagreements were resolved by discussion or confrontation with the third reviewer (L.C.). The selected full-text papers were screened using Citationchaser [26], an online tool developed for forward and backward citation chasing. The list of references and citations was downloaded and screened by one of the reviewers (M.D.S.) to identify any additional sources of information using the same two-step process and eligibility criteria.

### 2.3. Data Charting and Synthesis of the Results

Data were extracted from the papers included in the scoping review by two reviewers using a data extraction sheet developed by the reviewers and refined during the data charting process itself. The data extracted from the studies include specific details on the characteristics of the studies (year, journal and country of publication); the object of the identified measure, broadly categorised as interaction, relationship or attachment; the methods and tools used (e.g., observation/behavioural analysis, questionnaire/scales, etc.) with few details; the focus of the measure (i.e., whether it is on handler-dog or participant –dog, and if it is taken from the perspective of the handler, the animal or the DAI participant/recipient); the point of view (i.e., who is taking and analysing the measure: the experimenter/observer, the handler or the participant); and the general context, divided into DAI/DAI simulation contexts and tests or tasks involving therapy dogs. Graphical data are numerically and narratively synthesised and presented using figures, tables or graphs.

## 3. Results

### 3.1. Selection of the Sources of Evidence 

**Figure 1:**
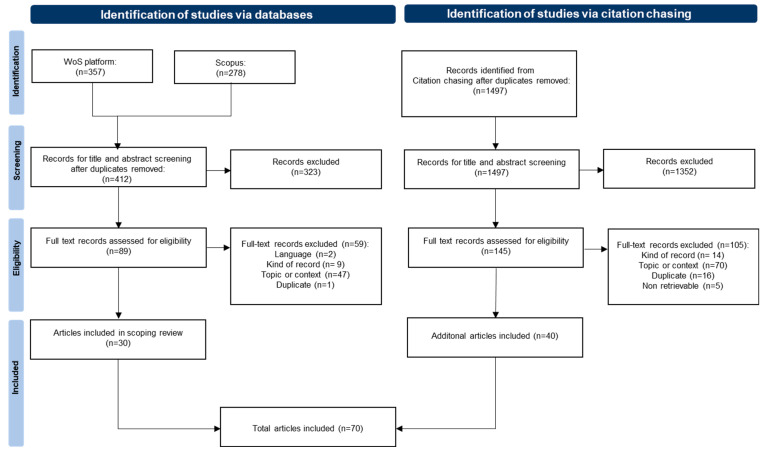
PRISMA flow diagram for record identification, screening, eligibility, and inclusion.

The PRISMA flow diagram [23] in Figure 1 shows the screening process. Of the 412 records initially identified via the database search, 30 were included. Forward and backward citation chasing from these included records resulted in the inclusion of further 40 records, with a total number of 70 records included for data charting. The data charting tool with all the included articles and extracted data is reported in Supplementary Materials (Table S1). 

### 3.2. Characteristics of the Sources of Evidence

**Figure 2:**
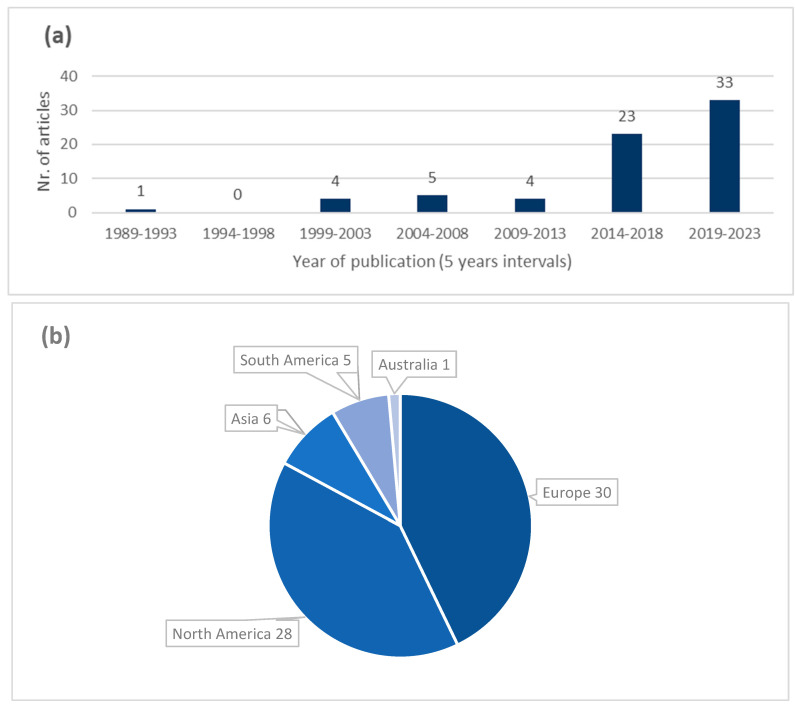
Distributions of the 70 selected records by years of publications (5 years intervals) (**a**) and geographical area (**b**).

Publication dates of included articles range from 1989 to today, with 80% of articles (*n* = 56) published in the last decade, as shown in Figure 2a. Most articles were from Europe (*n* = 30) and North America (*n* = 28) (Figure 2b), with the USA (*n* = 25), Italy (*n* = 12), Germany (*n* = 7), Argentina (*n* = 5) and Japan (*n* = 4) being the five countries where most of the included studies were conducted. The included articles were published by 38 different journals, with Table 2 listing the top publishing journals.

### 3.3. Measures and Instruments Identified

The identified measures are presented in two groups based on the overall context of the study. Most studies were conducted in the context of DAIs/DAI simulations (56 out of 70), while the remainder were considered separately because they report tests or tasks with therapy dogs in which aspects of relationship/bond/interaction with the participant or handler are considered (15 out of 70). The latter group includes a study reporting two online questionnaires also administered to therapy dog handlers [27]. It should be noted that one article [28] was counted in both categories, as it involves a test and a DAI simulation. All included studies, and their characteristics can be found in Supplementary Materials (Table S1). 

#### 3.3.1. DAI/DAI Simulation Contexts

Of the 56 studies conducted in the context of DAIs or simulations, 50 report some measure of the interaction and 6 of the bond. 

Table 3 lists the 56 studies specifying those based on observational or self-reporting methods or a combination thereof. The table also provides information on studies that employed live coding or video coding, as well as those that reported the calculation of inter-observer reliability or agreement.

The methods used to analyse the human-therapy dog interaction are mainly based on observational methods (*n* = 48 on 50 studies) and on behavioural analysis (e.g., frequency and/or duration of behaviours), in some cases using structured or semi-structured sheets, forms or working ethograms with categorisation, rating or scoring of interaction behaviours (e.g., [43,70,72]). Further details on the behaviours observed can be found in the Supplementary Materials (Table S1). 

Other specified tools are Observational Measurement of Engagement (modified) [31], Social Behaviour Observation Checklist [57,61], Behavioural Instrument for the Assessment of Dog Well-Being Before/During/After Therapy Sessions [69], OHAIRE coding system (Observation of Human-Animal Interaction for Research) [64,68], an evaluation form [29] and a checklist [37] of the interaction with the dog. Finally, interaction with the dog is also assessed using questionnaires and/or scales [30,33,34,39,40,42,74,75,76,77], including namely the Animal-assisted Therapy Flow Sheet [33,34,40] and the Human–Animal Interaction Scale (HAIS) [75].

Most of these studies (*n* = 31) consider the social interaction with the animal among the outcomes resulting from an intervention or from the presence of the therapy dog. Therefore, the focus is on the patient (or participant) who interacts with the animal. Nevertheless, other studies (*n* = 12) consider the dog’s perspective [28,30,35,44,52,53,54,56,58,62,65,69] or both perspectives (animal- and human-initiated interactions) (*n* = 8) [43,46,48,50,56,58,66,75].

As for the bond, four studies report the use of the Center for the Study of Animal Wellness Pet Bonding Scale (CSAW-PBS), a 28-item questionnaire used to assess the perceived bond between the participant and the therapy dog [80,81,82,83]; one study uses a modified version of the Lexington Attachment to Pets Scale (LAPS), referring to the therapy dog [79]; one study uses the OHAIRE coding system, already mentioned above, to derive a total score relating to human-animal bond [78]. All these instruments were used to analyse the bond between DAI participants and the therapy dogs and are self-administered (i.e., compiled by the participants themselves), with the only exception of OHAIRE, which is based on behaviour coding performed by the observer/experimenter [78]. 

#### 3.3.2. Tests or Tasks Involving Therapy Dogs

As for the 15 studies that report tests or tasks involving therapy dogs in which interaction, relationship or bond with the handler or participant of DAIs are analysed, most of them focus on the interaction. In detail, three studies analysed the behaviour of participants towards the therapy dog during a Trier Social Stress Test for Children (TSST-C) [84,85] and a test of preference of differential responsiveness [86], while the other nine focused on the therapy dog interacting with the handler/owner or experimenter. Table 4 reports the characteristics (of interest for this review), in particular, the details on the interaction measured and the kind of test performed in each of these studies. Finally, three studies report measures of dog-human bonding and relationship: one study analyses the therapy dog attachment style with the handler through a secure base test [28] and another study reports the assessment of the quality of dog-owner relationships during a test for DAIs suitability. This assessment is based on observations of eye contact between the dog and the owner and the dog’s compliance with the recall command [87]. The final study reports the administration of two online questionnaires to investigate the dog-owner relationship. Therapy dog owners/handlers formed part of the respondent population, and their responses were analysed and presented separately from the rest of the population, highlighting some different features of the dog-handler relationship when compared to the overall dog-owner population. The two questionnaires are the Cat/Dog–Owner Relationship Scale (C/DORS) and the LAPS [27].

#### 3.3.3. Mutual Interactions

While most of the measures are focused on one subject over the other, some measures were taken considering both perspectives of interacting subjects (9 out of 70 studies) [43,46,48,50,56,58,66,75,95]. In particular, Lee and colleagues [50] developed a pilot human-canine ethogram for an animal-assisted education program in which 51 behavioural items are identified from either the dog, the handler, or the participant. Another instrument used is the HAIS [75], a self-report instrument of 24 items used to describe and quantify behaviours performed by human and non-human animals during an episode of interaction. Furthermore, in a study of the still-face effect in therapy dogs, an effusiveness score was calculated contextually, in addition to the dog’s interaction behaviours (e.g., proximity, contact, looking, etc.), by observing the frequency with which the owner spoke to the dog, the tone of voice, and the intensity of petting [95]. Behavioural synchrony has also been studied in the handler-dog dyad [58] and between children and dogs during DAIs [66].

#### 3.3.4. The Dog Handler Connection

Within the included studies, 21 considered the interaction, relationship or bond between the therapy dog and the handler. 

In 8 of these studies, the dog-handler interaction is observed in the context of DAI/DAI simulations along with the interaction with the participants [35,44,50,52,54,56,62,69]. For example, in the dog ethogram reported by Corsetti et al. [44], behaviours such as looking at the handler or hiding behind the handler were considered. Another ethogram developed and reported by Lee and colleagues [50] lists 51 behavioural items identified from the dog, the handlers, and the participants during an animal-assisted education programme in primary schools. This ethogram includes items like looking at the handler (from the dog’s side), touching, looking, or talking to the dog (from the handler’s side). On the other hand, two studies report a questionnaire in which handlers assess their handling after the session (using a Likert scale) [77], or fill in a dog behavioural checklist of 31 items, including some items focused on the interaction with the handler and participant (e.g., depending excessively on the handler, or interacting in a friendly way) [30]. Additional details on the variables observed (when specified) can be found in Supplementary Materials (Table S1). The remaining 11 studies are focused primarily on the dog-handler dyad and its characteristics. For example, the study by Kujtkowska and colleagues [87] investigates the association between the dog-handler relationship/bond with a dog’s susceptibility to stress during a test for DAI suitability, and the canine-human relationship is graded on a scale considering eye contact between the dog and the owner and dog’s compliance with the recall command. Similarly, Wanser and Udell [28] assess therapy dog attachment style to their handler and its influence on the dog’s behaviour during a mock DAI session. The functioning of the dog-handler dyad is investigated during DAI, also by Pirrone and colleagues [58], who analyse social synchrony and stress in the dyad. The behaviours observed were gaze synchrony, joint attention, touch synchrony, dog responsiveness to the handler’s cue, and dog attention seeking. On the other hand, Kuzara et al. [49] distinguish different handler interaction styles through the observation of dog-directed handler behaviour (verbal and physical contact).

Moreover, as already pointed out, there are some studies analysing interaction behaviours of therapy dogs towards their owner/handlers during determinate tasks or tests, sometimes comparing therapy dogs to other dog populations (e.g., pet dogs) [88,92,93,94,95,96]. The behaviours observed in these studies are reported in Table 4. Finally, the therapy dog-handler relationship and bond have been investigated using the previously mentioned questionnaires C/DORS and LAPS [27].

## 4. Discussion

This scoping review aimed to investigate methods and measures used to assess the connection (i.e., bond, relationship and interaction) between dogs and humans in the context of DAIs. In particular, on the human side, it focused on the handler and the participant (i.e., recipient) of DAIs. 

The identified literature was primarily analysed in terms of what is measured (interaction, relationship or bond) and how it is measured (the methods or tools used), in addition to the general context (DAI or test situations with therapy dogs). Within the 70 research peer-reviewed articles included, the majority report the analysis of the interaction between the participant/recipient of the intervention and the therapy dog during DAIs, using behavioural analysis methods. However, it has been possible to identify some more structured tools that consider participant interactions with the therapy dog during DAIs, such as the OHAIRE coding system [64,68] and the Animal-assisted Therapy Flow Sheet [33,34,40]. In addition, the Behavioural Instrument for the Assessment of Dog Well-Being Before/During/After Therapy Sessions [69] analyses dog behaviour during DAIs, including interaction with the participant and handler, and the HAIS [75] considers both human and animal behaviours during the interaction. Instruments and tools for analysing the relationship and bond between therapy dogs and humans have also been identified, such as the CSAW-PBS [80,81,82,83] and a modified version of the LAPS [79]. These two instruments are filled in by AAI recipients, assuming their perspective of the relationship and bond with the therapy dog. On the other hand, the therapy dog-handler relationship and bond have been investigated through the administration of the questionnaires C/DORS and LAPS [27], while the dog’s attachment to his handler has been evaluated through a secure base test with subsequent classification into different attachment styles [28].

The recent review by Samet and colleagues [16], which updates the previous one by Wilson and Netting [15] on the status of instrument development in the field of human-animal interaction, raises some interesting considerations in the field of HAI measurement. First of all, as reported in the introduction, the authors highlight that “few tools were designed for HAI measurement in service, assistance, or working animal-human dyads” [16]. Although the differences in objectives, scope and methods with this review may point to some differences in results, the actual number of tools identified here is indeed small and not always species-specific or field-specific, but rather adapted from more general contexts. 

Another issue discussed by Samet and colleagues [16] relates to the reliability and validity of the measures. In particular, between face, content, criterion and construct validity, the authors argue the difficulty of assessing construct validity in HAI, which is complicated by terminological confusion in the field of HAI. In this review, measures were broadly categorised as measures of interaction, bond or relationship, but in some cases, these areas were not easy to distinguish. 

The discussion regarding the validity of the identified measures is also connected to a crucial issue in AAIs that requires further investigation: intervention fidelity. Intervention fidelity refers to the extent to which an intervention is implemented as intended. It is inevitably linked to the outcomes and effectiveness of the intervention (internal validity), as well as its replicability and generalizability (external validity) [97]. Rodriguez and colleagues [98] recently discussed fidelity, among other complexities in conducting AAI research, specifically referring to randomised controlled trials that are at the apex of the pyramid of evidence [98,99]. In their commentary, the authors report four facets of measuring fidelity: adherence to protocol, dosage, quality of intervention delivery, and participant responsiveness [98]. According to the authors, dosage refers not only to the frequency and duration of the intervention but, given the variety and complexity of the dynamics that occur during an AAI, ideally includes measures of the actual interactions that occur between humans and animals during the intervention [98]. The measures identified in this review can aid in describing the intervention and ensuring intervention fidelity.

Problems with construct validity can also arise when measures only consider the human perspective. In this review, a distinction was made between instruments that analysed interaction, relationship, and bond from the human’s perspective, the dog’s perspective or both. In particular, the dog’s perspective is considered in the more recent literature (last 10 years), often linked to welfare assessment of the dog involved in DAIs [28,30,35,44,48,52,54,56,58,62,65,69,87]. Although some early promoters of AAIs were already “passionately committed to welfare at both ends of the leash”, as properly pointed out by Peralta and Fine [100], it is only in recent years that the discourse on the role and welfare of animals involved in these interventions has evolved, along with efforts to standardise the sector. It is now recognised that animals involved in AAIs are like co-workers whose perspectives must be taken into account in order to maintain their welfare and motivation. Therefore, in addition to trying to avoid unnecessary stress for the animals, attempts are made to promote positive experiences and emotions, pursuing what is tautologically called ‘good welfare’. Despite the various facets of the ethical issues, good welfare (for both humans and animals) is considered particularly important in AAIs for the establishment of synergistic interactions. Beyond the moral standpoint, again quoting Peralta and Fine [100], the pursuit of good welfare is “the sustainable thing to do to empower a relationship that is so firmly supported on a strong human-animal bond”. In essence, the success of AAIs is inextricably linked to the human-animal relationship, which in turn is linked to both the welfare of the people involved and the welfare of the animals involved in the interventions [1,2]. 

A related and recent theme emerging from this review is the relationship between the therapy dog and the handler and how this influences the behaviour and welfare of the dog and the functioning of the dyad during the interventions [28,49,58,87]. Discussing the perspectives on attachment and bonding in the dog–human dyad, Payne and colleagues [7] highlighted the fact that relational factors between the dog and the owner or handler could affect dog performance. Therefore, it could be that certain attachment styles are beneficial in different contexts and could be tailored accordingly to improve the functionality of the dog-handler dyads [7]. Further knowledge of the functioning of the dog-handler dyad could thus allow for the correct matching of the dyad to a specific intervention and context. 

Finally, two recent studies were identified that investigated behavioural synchrony between therapy dogs and their handler or participants in DAIs [58,66]. Behavioural synchrony is defined as the coordination of behaviour between interacting partners and requires the perception and integration of multimodal communicative signals so that even in the case of dog-human interspecific interaction, it appears to be related to the affiliation between the partners [58,101]. In this sense, these measures can be a reference point to address the need to explore the mutual influences that occur in the dog-handler or dog-recipient interaction during DAIs [1,5].

### Limitations of the Study

The search strategy and eligibility criteria for this review were also defined on the basis of the available resources. The inclusion of only research articles published in peer-reviewed journals and the exclusion of other sources such as books, book chapters, abstracts, and grey literature may have limited the number of results. Another limitation relates to the inclusion criteria for the methods and instruments used to assess the interaction, relationship or bond between the therapy dog and the handler or DAI participant. In order to apply a consistent inclusion criterion, we included both articles focused primarily on the relevant measures and articles that only reported them as collateral information. Furthermore, some of these interaction measures may not have been included because the keywords did not appear in the title, abstracts or keywords and were therefore not detected by literature search. 

## 5. Conclusions

This scoping review examined the measures and tools used to assess human-therapy dog interaction, relationship and bonding in the context of DAIs. The tools and methods identified provide an up-to-date, state-of-the-art scenario in the field and can be used by both practitioners and researchers to further investigate aspects of human-dog interaction in the field of DAIs. Despite the central role of the dog-human relationship and interaction in the field of DAIs, relatively few structured tools are available to assess interaction, relationship and bond. As advocated by other authors [15,16], these instruments need to be used and further developed to consolidate their validity and reliability, with the aim of identifying solid, common instruments based on a common language. The search for measures of mutual interaction and tools that take the animal’s perspective into account, as well as tools for analysing the dog-handler dyad, is a growing area of research. Given the interconnectedness of the human-animal relationship and well-being, can we identify measures of the relationship that predict human and animal well-being during AAIs or the effectiveness of the intervention? The answer can open new, exciting research perspectives in the field of AAIs.

## Figures and Tables

**Table 1:** Search query used in the Web of Science Platform.

Web of Science Platform (Core Collection, Medline, SCIelo Citation Index)
(TS = ((((animal* OR dog* OR canine*) NEAR/0 assisted NEAR/0 (intervention* OR activit* OR education OR therap*)) OR ((aai OR aat OR aae OR aaa) NEAR/5 (dog)) OR “pet*therapy” OR “therapy*dog”))) AND TS = (((((dog* OR pet* OR animal* OR Cani*) NEAR/10 (human* OR handler* OR owner* OR patient* OR user* OR child*)) NEAR/10 (interaction* OR relation* OR bond)) AND (measure* OR test OR assess* OR survey OR indicator* OR scale OR score)))

**Table 2:** Top journals publishing three or more articles on the topic of interest of this review (*n* = 70).

Journal Name	Number of Articles (%)
Anthrozoös	9 (13%)
Animals	8 (11%)
Journal of Veterinary Behavior	7 (10%)
Applied Animal Behaviour Science	5 (7%)
Psychogeriatrics	3 (4)

**Table 3:** Methods used in the 56 studies conducted in the context of DAIs/DAIs simulations.

What Is Measured	Methods	Live Coded vs. Video Coded	Number of Articles	References	Inter-Observer Reliability/Agreement	References
Interaction	Observational methods	Live coded	18	[29,30,31,32,33,34,35,36,37,38,39,40,41,42,43,44,45,46]	8	[31,32,33,34,35,39,40,42]
		Video coded	25	[28,47,48,49,50,51,52,53,54,55,56,57,58,59,60,61,62,63,64,65,66,67,68,69,70]	21	[28,48,49,50,51,52,53,54,55,56,57,58,60,61,63,64,65,66,67,68,69] ^1^
		Combination	2	[71,72]	1	[72]
		Not specified	1	[73]	1	[73]
	Self-reported	\	2	[74,75] ^2^	\	\
	Combination of methods (observational and self-reporting)	Live coded (observational methods)	2	[76,77]	\	\
Bond	Observational methods	Video coded	1	[78]	1	[78]
	Self-reported	\	5	[79,80,81,82,83]	\	\

^1^ Glenk et al. [65] assessed intra-observer reliability. ^2^ In Dell et al. [74], the questionnaires may also denote the bond.

**Table 4:** Test/task involving therapy dogs, measures of interaction used and relative references.

Object of Study in Therapy Dogs	Test/Task in Which Interaction Is Analysed	Measure of Interaction	Ref. ^1^
Sustained attention to the owner ^2^	Baseline attention and selective attention test	Length of uninterrupted gazes and frequency of gaze shifting	[88]
Sociocognitive abilities	Sociability and gazing tests	Duration of time close and physical contact (sociability test) and gazing duration (gazing test)	[89]
Persistence in learned responses	Gazing tasks	Gazing time	[90]
Problem solving	Problem solving task	Gazing frequency	[91]
Showing behaviour	Showing task	Behaviours towards the owner ^2^: gazing, gaze alternation, other behaviours	[92]
Behaviour in help request	Unsolvable task	Gazing time, latency and direction of first gaze, frequency of gaze alternation, contact with the people	[93]
Personality and cognitive profiles during unsolvable task		Looking behaviour: looking overall, referential looking	[94]
Effect of still face	Still face test	Affiliative behaviours (proximity, contact, gazing, licking) and presence of begging behaviours ^3^	[95]
Factors Contributing to Successful Spontaneous Dog-Human Cooperation	Out-f-reach task	Attentiveness (closeness, orienting, gazing)	[96]

^1^ Ref. = reference; ^2^ in most cases, the handler is the owner of the therapy dog. Therefore, in the context of this review, they are to be considered synonymous; ^3^ in this study, the owner’s effusiveness is scored as well (frequency in which the owner speaks to the dog, tone of voice, intensity of petting).

## Data Availability

No new data were created or analyzed in this study. Data sharing is not applicable to this article. The PRISMA-ScR protocol is available from the authors upon request.

## Supplementary Materials

The following supporting information can be downloaded at: https://www.mdpi.com/article/10.3390/ani14030410/s1. Table S1: data charting of included articles.
