# Supplementary material for: How to Measure Human-Dog Interaction in Dog Assisted Interventions? A Scoping Review
Source: Animals (Basel). 2024 Jan 26;14(3):410. doi: 10.3390/ani14030410 (PMC10854530; doi:10.3390/ani14030410)
Supplement: Supplementary file 1 [file animals-14-00410-s001.zip › animals-2809704-supplementary-final version.pdf]

Table S1: data charting of included articles

ShortTitle	Title	Journal	Country	Geographical region	What is measured	Kind of measure	How is measured	Focus	Point of view	Context
**Ambrosi (2019)**	Randomized controlled study on the effectiveness of animal-assisted therapy on depression, anxiety, and illness perception in institutionalized elderly	Psychogeriatrics	Italy	Europe	Interaction	Observation /Behavioural analysis	Frequency of verbal (speaking, sounds, vocalizations) and nonverbal (petting, stroking, throwing food or toy) interactions towards the dog	Participant > dog	Observer/experimenter	DAI/DAI simulation
**Ávila-Álvarez (2020)**	Improving social participation of children with autism spectrum disorder: Pilot testing of an early animal-assisted intervention in Spain	Health and Social Care in the Community	Spain	Europe	Interaction	Questionnaire/Scale	AAT Flow Sheet (9 items) includes frequency of child–dog social relationships (6 items: looked at dog, touched dog, spoke to dog, remembered and used dog's name, engaged in activity with dog, reminisced about own dog). Likert-type responses	Participant > dog	Observer/experimenter	DAI/DAI simulation
**Banks (2008)**	Animal-Assisted Therapy and Loneliness in Nursing Homes: Use of Robotic versus Living Dogs	Journal of the American Medical Directors Association	USA	North America	Bond	Questionnaire/Scale	Lexington Attachment to Pets Scale (LAPS) modified (MLAPS)	Participant > dog	Participant	DAI/DAI simulation
**Beetz (2012)**	Effects of social support by a dog on stress modulation in male children with insecure attachment	Frontiers in Psychology	Germany and Austria	Europe	Interaction	Observation /Behavioural analysis	Behaviours of the participant and his interaction with the dog. Frequencies and/or durations, 49 variables considered	Participant > dog	Observer/experimenter	TEST Trier Social Stress Test for Children (TSST-C)
**Berry (2012)**	Developing effective animal-assisted intervention programs involving visiting dogs for institutionalized geriatric patients: A pilot study	Psychogeriatrics	Italy	Europe	Interaction	Observation /Behavioural analysis	Occurrences of activities and social interactions with the dog (ethogram), coded as spontaneous/mediated by the handler. Interactions are: "looking at", "talking to" or touching the dog. Also frequency of smile episodes and their co-occurrence with social interactions	Participant > dog	Observer/experimenter	DAI/DAI simulation
**Bidoli (2022)**	Dogs working in schools– Safety awareness and animal welfare	Journal of Veterinary Behavior	Germany	Europe	Interaction	Observation /Behavioural analysis	Ethogram of the dog with general physical activity behaviour measured in duration of occurrence (state) and more specific behaviour, divided into a dozen of categories (including interaction with the participant),	Dog <> participant (and teacher)	Observer/experimenter	DAI/DAI simulation

							documented in terms of duration of occurrence (state) or frequency (event). Interaction of the participant with the dog registered as well. Moreover, each behaviour (both from canine and human part) scored with 1 point and assigned to 1 of the following categories: "Critical"; "Problematic"; "Innocuous". Detailed description of the interactions considered in the article			
**Caprilli (2006)**	Animal-assisted activity at A. Meyer children's hospital: A pilot study	Evidence-based Complementary and Alternative Medicine	Italy	Europe	Interaction	Questionnaire/Scale	Scale n.1 (child–animal interaction): 9 items (brushing; ordering the dog; petting; playing; nourishing; talking; taking; walking; watching) with 4 scores (active, active if solicited, resistant, passive)	Participant > dog	Observer/experimenter	DAI/DAI simulation
**Carballo (2020)**	Assistance and Therapy Dogs Are Better Problem Solvers Than Both Trained and Untrained Family Dogs	Frontiers in Veterinary Science	Hungary	Europe	Interaction	Observation/Behavioural analysis	Grazing frequency	Dog > participant/experimenter	Observer/experimenter	TEST problem solving task
**Cavalli (2018)**	Are animal-assisted activity dogs different from pet dogs? A comparison of their sociocognitive abilities	Journal of Veterinary Behavior	Argentina	South America	Interaction	Observation/Behavioural analysis	Duration of time close and physical contact (sociability test) and gazing duration (gazing test)	Dog > experimenter	Observer/experimenter	TEST 3 behavioural tasks (sociability test, gazing test, and A-not-B task)
**Cavalli (2019)**	Persistence in learned responses: A comparison of Animal Assisted Intervention and pet dogs	Journal of Veterinary Behavior	Argentina	South America	Interaction	Observation/Behavioural analysis	Gazing time in gazing test	Dog > experimenter	Observer/experimenter	TEST two sociocognitive tasks (gazing and object choice) and a nonsocial task (problem-solving)
**Cavalli (2020)**	Gazing as a help requesting behavior: a comparison of dogs participating in animal-assisted interventions and pet dogs	Animal Cognition	Argentina	South America	Interaction	Observation/Behavioural analysis	Gazing time, latency and direction of first gaze, frequency of gaze alternation, contact with the people	Dog > handler/owner and experimenter	Observer/experimenter	TEST unsolvable task

**Cavalli (2020)**	Showing behavior in Animal Assisted Intervention and pet dogs	Behavioral processes	Argentina	South America	Interaction	Observation /Behavioural analysis	Behaviours towards the owner: gazing, gaze alternation, other behaviours	Dog > handler/ owner	Observer/ experimenter	TEST showing task
**Cavalli (2023)**	Still-face effect in domestic dogs: comparing untrained with trained and animal assisted interventions dogs	Learning & Behavior	Argentina	South America	Interaction	Observation /Behavioural analysis	Affiliative behaviours (proximity, contact, gazing, licking) and presence of begging behaviours. Owner's effusiveness: frequency in which owner spoke to the dog, tone of voice, intensity of petting	Dog <> handler/ owner	Observer/ experimenter	TEST Still face test
**Chubak (2017)**	Pilot Study of Therapy Dog Visits for Inpatient Youth With Cancer	Journal of Pediatric Oncology Nursing	USA	North America	Interaction	Observation /Behavioural analysis	Semi structured form developed for the study considering also interactions with the dog such as petting, playing, brushing, hugging, talking, watching, going out of bed to interact with the dog, dog getting onto bed or onto patient's lap, other	Participant <> dog	Observer/ experimenter	DAI/DAI simulation
**Corsetti (2019)**	Evaluating Stress in Dogs Involved in Animal-Assisted Interventions	Animals	Italy	Europe	Interaction	Observation /Behavioural analysis	Dog ethogram of 53 behavioural patterns gathered into 6 categories, with some interaction behaviours (e.g. looking, requesting attention). Details of the ethogram in the Appendix of the article.	Dog > participant/handler	Observer/ experimenter	DAI/DAI simulation
**Csepregi (2023)**	Factors Contributing to Successful Spontaneous Dog-Human Cooperation	Animals	Hungary	Europe	Interaction	Observation /Behavioural analysis	Attentiveness (closeness, orienting at owner, gazing)	Dog > handler/ owner	Observer/ experimenter	TEST ‘out–of–reach’ task
**Dell (2019)**	Animal-assisted therapy in a Canadian psychiatric prison	International Journal of Prisoner Health	Canada	North America	Interaction/bond	Questionnaire/Scale	Likert rating of the experience with the dog - perceived interaction (also measure of bond)	Participant > dog	Participant	DAI/DAI simulation
**Friedmann (2019)**	Relationship of Behavioral Interactions during an Animal-assisted Intervention in Assisted Living to Health-related Outcomes	Anthrozoös	USA	North America	Interaction	Observation /Behavioural analysis	Percent of sessions the participant attended in which occurred looking at, touching, brushing, talking to, walking with, and giving treats to the dog	Participant > dog	Observer/ experimenter	DAI/DAI simulation
**Funahashi (2014)**	Brief Report: The Smiles of a Child with Autism Spectrum Disorder During an Animal-assisted Activity May Facilitate Social Positive Behaviors—	Journal of Autism and Developmental Disorders	Japan	Asia	Interaction	Observation /Behavioural analysis	Smiles, positive social behaviours (watching the dog, voluntarily touching the dog, dog coming up to the knee, holding the dog, verbal communication with the dog) and negative social behaviours (escaping,	Participant > dog	Observer/ experimenter	DAI/DAI simulation

	Quantitative Analysis with Smile-detecting Interface						outside of the circle, anxiety and anger)			
**Gee (2015)**	Does Physical Contact with a Dog or Person Affect Performance of a Working Memory Task?	Anthrozoös	USA	North America	Interaction	Questionnaire/Scale	11 item self-report dog evaluation form: comfort, discomfort, ambivalence, desire to interact with the dog + experimenter rating of participant's comfort level with the dog (Likert scale)	Participant > dog	Participant and experimenter	DAI/DAI simulation
**Germone (2019)**	Animal-assisted activity improves social behaviors in psychiatrically hospitalized youth with autism	Autism	USA	North America	Interaction	Observation /Behavioural analysis with coding system	Timed interval coding tool OHAIRE - considers also interactions with animals, e.g. talk, gesture, look, touch, affection, prosocial behaviour	Participant > dog	Observer/ experimenter	DAI/DAI simulation
**Glenk (2014)**	Salivary cortisol and behavior in therapy dogs during animal-assisted interventions: A pilot study	Journal of Veterinary Behavior	Austria	Europe	Interaction	Observation /Behavioural analysis	Dog behaviour - Frequency and durations, including response to human action (takes a treat/obeys to command)	Dog > participant	Observer/ experimenter	DAI/DAI simulation
**Griffioen (2019)**	Changes in behavioural synchrony during dog-assisted therapy for children with autism spectrum disorder and children with Down syndrome	Journal of Applied Research in Intellectual Disabilities (JARID)	The Netherlands	Europe	Interaction	Observation /Behavioural analysis	Coding of movement direction of the participant and the dog. Synchronous movement patterns of participants and dogs. Time series of the participants and dog's movement directions subjected to cross-recurrence quantification analysis	Participant <> dog	Observer/ experimenter	DAI/DAI simulation
**Grigore (2014)**	Interaction with a Therapy Dog Enhances the Effects of Social Story Method in Autistic Children	Society and Animals	Romania	Europe	Interaction	Observation /Behavioural analysis	Frequency of appropriate and initiated social interactions and level of prompt needed. Initiated social interactions include approaching the dog	Participant > dog	Observer/ experimenter	DAI/DAI simulation
**Guérin (2018)**	Reliability and Validity Assessment of the Observation of Human-Animal Interaction for Research (OHAIRE) Behavior Coding Tool	Frontiers in Veterinary Science	USA	North America	Interaction	Observation /Behavioural analysis with coding system	Timed interval coding tool OHAIRE - considers also interactions with animals, e.g. talk, gesture, look, touch, affection, prosocial behaviour	Participant > dog	Observer/ experimenter	DAI/DAI simulation
**Hill (2023)**	Investigating Dog Welfare When Interacting with Autistic Children within Canine-Assisted Occupational Therapy Sessions: A Single Case Study	Animals	Australia	Australia	Interaction	Observation /Behavioural analysis with tool/checklist	Behavioural Instrument for the Assessment of Dog Well-Being Before/During/After Therapy Sessions, checklist of ten items including therapy dog's interaction with people. Each item scored 0-3 depending on the severity of the behaviour.	Dog > participant/handler	Observer/ experimenter	DAI/DAI simulation

**Holt (2015)**	Animal Assisted Activity with Older Adult Retirement Facility Residents: The PAWSitive Visits Program	Activities, Adaptation & Aging	USA	North America	Bond	Questionnaire/Scale	Center for the Study of Animal Wellness Pet Bonding Scale (CSAW-PBS)	Participant > dog	Participant	DAI/DAI simulation
**Kaiser (2002)**	A Dog and a "Happy Person" Visit Nursing Home Residents	Western Journal of Nursing Research	USA	North America	Interaction	Observation/Behavioural analysis	Frequencies and/or durations. Behaviours initiated by participant: nonverbal behaviours included shaking hands, patting, disengaging, affection, moving closer, and commands. Behaviours initiated by the visitor (dog): shaking paw/hands, patting/petting, disengaging, and moving closer	Participant <> dog	Observer/experimenter	DAI/DAI simulation
**Kline (2020)**	Randomized Trial of Therapy Dogs Versus Deliberative Coloring (Art Therapy) to Reduce Stress in Emergency Medicine Providers	Academic Emergency Medicine	USA	North America	Interaction	Observation/Behavioural analysis with evaluation form	Interaction with the dog: touch (Yes or No); Grade the interaction with the dog (Likert 1-5); time of interaction	Participant > dog	Handler/observer	DAI/DAI simulation
**Koda (2015)**	Stress levels in dogs, and its recognition by their handlers, during animal-assisted therapy in a prison	Animal Welfare	Japan	Asia	Interaction	Questionnaire/Scale	Behavioural items in 3 behavioural categories (stressful, relaxed, positive emotional) some of which focused on the interaction with handler and participant, e.g. depending excessively on the handler, interacting in a friendly way	Dog > participant/handler and other members	Dog owner/handler	DAI/DAI simulation
**Koda (2015)**	Effects of a Dog-assisted Program in a Japanese Prison	Asian Journal of Criminology	Japan	Asia	Interaction	Questionnaire/Scale	Questionnaires to evaluate participant-dog interaction (tenderness toward the dogs, quantity of talking, willingness to have contact with the dogs, interest in the dogs and ease of interaction) and handler-dog interaction (handling)	Participant/handler > dog	Dog owner/handler	DAI/DAI simulation
**Koda (2016)**	Effects of a Dog-Assisted Intervention Assessed by Salivary Cortisol Concentrations in Inmates of a Japanese Prison	Asian Journal of Criminology	Japan	Asia	Interaction	Questionnaire/Scale	Questionnaire on interactional skills of inmates (Likert scale)	Participant > dog	Dog owner/handler	DAI/DAI simulation
**Kramer (2009)**	Comparison of the effect of human interaction, animal-assisted therapy, and AIBO-assisted therapy on long-	Anthrozoös	USA	North America	Interaction	Observation/Behavioural analysis	Conversation, touch, looking at others, hand gestures, smiles and laughs (with different categories each), and duration of looks. Total attention obtained summing frequency of	Participant > dog	Observer/experimenter	DAI/DAI simulation

	term care residents with dementia						interaction behaviours (conversation, look, touch and social gestures)			
**Kujtkowska (2020)**	A pilot study on the qualitative assessment of the impact of human-canine relationships on dogs' susceptibility to stress[1]	Dog Behavior	Poland	Europe	Relationship/bond	Observation /Behavioural analysis	Eye contact between the dog and the owner and dog's compliance with the recall command (grading good, average, poor)	Dog > handler/ owner	Observer/ experimenter	TEST for AAI suitability
**Kuzara (2019)**	Exploring the Handler-Dog Connection within a University-Based Animal-Assisted Activity	Animals	USA	North America	Interaction	Observation /Behavioural analysis	Classification in handler interaction styles. Duration and frequency of dog-directed handler behaviour (i.e. verbal and physical contact). Two dimensions of handlers' dog-directed behaviour emerged (e.g., warmth, control) revealing distinct handler interaction styles	Handler > dog	Observer/ experimenter	DAI/DAI simulation
**Lee (2022)**	Development of a pilot human-canine ethogram for an animal-assisted education programme in primary schools – A case study	Applied Animal Behaviour Science	China	Asia	Interaction	Observation /Behavioural analysis	Human-animal interaction ethogram of 51 behavioural items identified from the dog, the handlers, and the students (participants). Full ethogram reported in the article	Dog <> handler <> participant	Observer/ experimenter	DAI/DAI simulation
**Martin (2002)**	Animal-Assisted Therapy for Children with Pervasive Developmental Disorders	Western Journal of Nursing Research	USA	North America	Interaction	Observation /Behavioural analysis	Frequencies and/or durations of interactions (behaviours and verbal), in some cases towards the dog (e.g. touching, giving treats, looking at, talk to)	Participant > dog	Observer/ experimenter	DAI/DAI simulation
**Marx (2010)**	The Impact of Different Dog-related Stimuli on Engagement of Persons With Dementia	American Journal of Alzheimer's Disease & Other Dementias	USA	North America	Interaction	Observation /Behavioural analysis with tool/checklist	Observational Measurement of Engagement (modified): duration of engagement and attitude toward the stimulus (dog)	Participant > dog	Observer/ experimenter	DAI/DAI simulation
**McCullough (2018)**	Physiological and behavioral effects of animal-assisted interventions on therapy dogs in pediatric oncology settings	Applied Animal Behaviour Science	USA	North America	Interaction	Observation /Behavioural analysis	Dog ethogram of 26 behaviours divided into three categories: affiliative, moderate stress, and high-stress indicators, with some interaction behaviours (leaning or resting body or head against a person	Dog > participant or handler	Observer/ experimenter	DAI/DAI simulation

[1] This study was included even if reporting a qualitative assessment as it grades on a scale (good, average, poor) the human-canine relationship, based on observations of eye contact between the dog and the owner, and the dog's compliance with the recall command.

							or object, licking a person…). Full ethogram reported in the article			
**Mezza (2022)**	Process Evaluation of Animal Assisted Therapies with Children: The Role of the Human-Animal Bond on the Therapeutic Alliance, Depth of Elaboration, and Smoothness of Sessions	Mediterranean Journal of Clinical Psychology	Italy	Europe	Bond	Observation /Behavioural analysis with coding system	OHAIRE: Interval behaviour coding to note the presence or absence of a behaviour during 10-second interval. Interactive Behaviours (6 categories of social and communication behaviours directed to adults and animals, namely talking, looking, gesturing, touching, showing affection, and being prosocial); Emotional Display; Interfering Behaviours. Total score relating to the human-animal bond considered: scale (1-6) calculated within the "interactive behaviours" category and obtained by rating the presence or absence of any social interactive behaviours toward the animal.	Participant > dog	Observer/ experimenter	DAI/DAI simulation
**Mongillo (2015)**	Validation of a selection protocol of dogs involved in animal-assisted intervention	Journal of Veterinary Behavior	Italy	Europe	Interaction	Observation /Behavioural analysis	Time of interaction and percentage of time of negative interactions (the dog avoids the stranger trying to interact or the dog freezes and stiffens while the stranger interacts)	Dog > participant/experimenter	Observer/ experimenter	DAI/DAI simulation
**Mongillo (2017)**	Sustained attention to the owner is enhanced in dogs trained for animal assisted interventions	Behavioural Processes	Italy	Europe	Interaction	Observation /Behavioural analysis	Length of uninterrupted gazes and frequency of gaze shifting	Dog > handler/ owner and participant/experimenter	Observer/ experimenter	TEST baseline attention and selective attention
**Ng (2014)**	The effect of dog–human interaction on cortisol and behavior in registered animal-assisted activity dogs	Applied Animal Behaviour Science	USA	North America	Interaction	Observation /Behavioural analysis	Ethogram of dog behaviours while petted by the handler (interaction item: licking person)	Dog > handler/ participant	Observer/ experimenter	DAI/DAI simulation
**Olsen (2019)**	Engagement in elderly persons with dementia attending animal-assisted group activity	Dementia	Norway	Europe	Interaction	Observation /Behavioural analysis	Ethogram. Frequency and duration of interactions with the dog, e.g. conversation, looking, touch, smile, …	Participant > dog	Observer/ experimenter	DAI/DAI simulation

**Palestrini (2017)**	Stress level evaluation in a dog during animal-assisted therapy in pediatric surgery	Journal of Veterinary Behavior	Italy	Europe	Interaction	Observation /Behavioural analysis	Dog interaction with the child, with the handler, with the people, withdrawal (duration). Children's (participant) interactions with the dog recorded in terms of duration of occurrence (states)	Dog > participant/handler and participant > dog	Observer/ experimenter	DAI/DAI simulation
**Pérez-Sáez (2019)**	Effects of Dog-Assisted Therapy on Social Behaviors and Emotional Expressions: A Single-Case Experimental Design in 3 People With Dementia	Journal of Geriatric Psychiatry and Neurology	Spain	Europe	Interaction	Observation /Behavioural analysis with tool/checklist	Social Behaviour Observation Checklist (SBOC): smile/laugh, looks, physical contact, leans-toward stimulus, and verbalizations towards therapist or dog. Sum of all the social behaviours coded in each session calculated and used as a total score.	Participant > dog	Observer/ experimenter	DAI/DAI simulation
**Phelps (2008)**	An investigation of the effects of dog visits on depression, mood, and social interaction in elderly individuals living in a nursing home	Behavioral Interventions	USA	North America	Interaction	Observation /Behavioural analysis	Percentage of intervals in which each participant engages in verbal/nonverbal interactions with the dog	Participant > dog	Observer/ experimenter	DAI/DAI simulation
**Piotti (2021)**	Personality and Cognitive Profiles of Animal-Assisted Intervention Dogs and Pet Dogs in an Unsolvable Task	Animals	Italy	Europe	Interaction	Observation /Behavioural analysis	Looking behaviour: looking overall, referential looking	Dog > handler/ owner	Observer/ experimenter	TEST unsolvable test
**Pirrone (2017)**	Measuring social synchrony and stress in the handler-dog dyad during animal-assisted activities: A pilot study	Journal of Veterinary Behavior	Italy	Europe	Interaction	Observation /Behavioural analysis	Social synchrony: gaze synchrony, joint attention, and touch synchrony/responsiveness to handler's cue/attention seeking handler or patient/spontaneous physical contact seeking with patients	Dog <> handler and dog > participant	Observer/ experimenter	DAI/DAI simulation
**Prothmann (2015)**	Analysis of child—dog play behavior in child psychiatry	Anthrozoös	Germany	Europe	Interaction	Observation /Behavioural analysis	Multistage coding system (4 categories: posture, visual contact, behaviour towards dog, behaviours towards handler). Behaviours towards the dog: calling/talking, stroking, hugging/cuddling, invitation to play, distancing	Participant > dog	Observer/ experimenter	DAI/DAI simulation
**Prothmann (2015)**	Preference for, and responsiveness to, people, dogs and objects in children with autism	Anthrozoös	Germany	Europe	Interaction	Observation /Behavioural analysis	Multistage category system and coding: interaction with the dog (initiation or response: talking to, physical contact, offer to play, response to dog's offer to play)	Participant > dog	Observer/ experimenter	TEST of preference of differential responsiveness

**Redefer (1989)**	Brief report: Pet-facilitated therapy with autistic children	Journal of Autism and Developmental Disorders	USA	North America	Interaction	Observation/Behavioural analysis	Social interaction: any instance of verbal or nonverbal behaviour directed to the therapist or dog	Participant > dog	Observer/experimenter	DAI/DAI simulation
**Richeson (2003)**	A therapeutic recreation intervention using animal-assisted therapy: effects on the subjective well-being of older adults	Annual in Therapeutic Recreation	USA	North America	Interaction	Questionnaire/Scale	AAT Flow Sheet (9 items) includes frequency of child–dog social relationships (6 items: looked at dog, touched dog, spoke to dog, remembered and used dog's name, engaged in activity with dog, reminisced about own dog). Likert-type responses	Participant > dog	Handler and/or observer	DAI/DAI simulation
**Richeson (2003)**	Effects of animal-assisted therapy on agitated behaviors and social interactions of older adults with dementia	American Journal of Alzheimer's Disease & Other Dementias	USA	North America	Interaction	Questionnaire/Scale	AAT Flow Sheet (9 items) includes frequency of child–dog social relationships (6 items: looked at dog, touched dog, spoke to dog, remembered and used dog's name, engaged in activity with dog, reminisced about own dog). Likert-type responses	Participant > dog	Observer/experimenter	DAI/DAI simulation
**Riggio (2021)**	The dog–owner relationship: Refinement and validation of the Italian c/dors for dog owners and correlation with the laps	Animals	Italy	Europe	Relationship	Questionnaire/Scale	C/DORS and LAPS	handler/owner > dog	Dpg owner/handler	Online questionnaire
**Robino (2021)**	College Student Mental Health in an Animal-Assisted Intervention Program: A Preliminary Study	Journal of Creativity in Mental Health	USA	North America	Interaction	Questionnaire/Scale	Human–Animal Interaction Scale (HAIS): self-report instrument of 24 items to describe and quantify behaviours performed by human and non-human animals during an episode of interaction. Rating with Likert scale, behaviours performed by humans and by animals. Total score: the higher, the greater quantity of positive human animal interactions.	Dog <> participant	Participant	DAI/DAI simulation
**Robino (2022)**	Sustained Effects of Animal-Assisted Crisis Response on Stress in School Shooting Survivors	Human Animal Interaction Bulletin	USA	North America	Bond	Questionnaire/Scale	Center for the Study of Animal Wellness Pet Bonding Scale (CSAW-PBS)	Participant > dog	Participant	DAI/DAI simulation
**Rodrigo-Claverol (2023)**	Human–Animal Bond Generated in a Brief Animal-Assisted Therapy Intervention in Adolescents	Animals	Spain	Europe	Bond	Questionnaire/Scale	Center for the Study of Animal Wellness Pet Bonding Scale (CSAW-PBS)	Participant > dog	Participant	DAI/DAI simulation

	with Mental Health Disorders								
**Sarrafchi (2022)**	Effect of human-dog interaction on therapy dog stress during an on-campus student stress buster event	Applied Animal Behaviour Science	Canada	North America	Interaction	Observation /Behavioural analysis	Dog ethogram: Interaction with participant (approach or avoid) and owner (interact)	Dog > participant/handler/owner	Observer/experimenter	DAI/DAI simulation
**Sellers (2006)**	The evaluation of an animal assisted therapy intervention for elders with dementia in long-term care	Activities, Adaptation & Aging	USA	North America	Interaction	Observation /Behavioural analysis with tool/checklist	Social Behaviour Observation Checklist (SBOC): smile/laugh, leans, looks, touch, and verbalization.	Participant > dog	Observer/experimenter	DAI/DAI simulation
**Thodberg (2016)**	Behavioral Responses of Nursing Home Residents to Visits From a Person with a Dog,a Robot Seal or aToy Cat	Anthrozoös	Denmark	Asia	Interaction	Observation /Behavioural analysis	Frequency and duration of physical contact with the animal, talk directed to the animal and the visiting person, and visual contact with the animal or the visiting person. Ethogram reported in the article	Participant > dog	Observer/experimenter	DAI/DAI simulation
**Thodberg (2021)**	Dog visits in nursing homes-increase complexity or keep it simple? A randomised controlled study	Plos One	Denmark	Europe	Interaction	Observation /Behavioural analysis	Frequency and duration of physical contact and talk directed to the dog	Participant > dog	Observer/experimenter	DAI/DAI simulation
**Townsend (2022)**	Reactivation of a Hospital-Based Therapy Dog Visitation Program during the COVID-19 Pandemic	Animals	USA	North America	Interaction	Observation /Behavioural analysis with tool/checklist	Checklist with interactions classified in 3 categories: duration of talk, pet, talk and pet. Total interaction time calculated	Participant > dog	Observer/experimenter	DAI/DAI simulation
**Turner-Collins (2019)**	Does Anthropomorphism of Dogs Affect Pain Perception in Animal-Assisted Interventions? An Exploratory Study	Anthrozoös	USA	North America	Bond	Questionnaire/Scale	Center for the Study of Animal Wellness Pet Bonding Scale (CSAW-PBS)	Participant > dog	Participant	DAI/DAI simulation
**Uccheddu (2018)**	Assessing behavior and stress in two dogs during sessions of a reading-to-a-dog program for children with pervasive developmental disorders	Dog behavior	Italy	Europe	Interaction	Observation /Behavioural analysis	Frequency and/or duration of 18 dog behaviours, including attention seeking (participant), avoidance (participant), looking at participant or handler	Dog > participant/handler	Observer/experimenter	DAI/DAI simulation

**Walters Esteves (2008)**	Social effects of a dog's presence on children with disabilities	Anthrozoös	Florida	North America	Interaction	Observation /Behavioural analysis	Interactions categorized as positive/negative/verbal/non verbal/initiated/prompted. Within positive non-verbal, for example: touching the dog by petting, throwing/handing treats to the dog, holding the leash, or walking the dog	Participant > dog	Observer/ experimenter	DAI/DAI simulation
**Wanser (2019)**	Does attachment security to a human handler influence the behavior of dogs who engage in animal assisted activities?	Applied Animal Behaviour Science	USA	North America	Bond + interaction	Observation /Behavioural analysis	Attachment style classification (secure base test) + Behavioural states: gazing at the participant, touching the participant, participant proximity-seeking, gazing at the handler, touching the handler, and handler proximity-seeking	Dog > handler/ participant	Observer/ experimenter	TEST secure base test + DAI/DAI simulation
**Wedl (2015)**	Children with Avoidant or Disorganized Attachment Relate Differently to a Dog and to Humans During a Socially Stressful Situation	Anthrozoös	Germany	Europe	Interaction	Observation /Behavioural analysis	Behavioural parameters coded "physical contact with the dog" (stroking, touching, holding the dog), "playing with the dog," "talking to the dog"	Participant > dog	Observer/ experimenter	TEST Experimental Trier Social Stress Test for Children
**Wesenberg (2019)**	Effects of an animal-assisted intervention on social behaviour, emotions, and behavioural and psychological symptoms in nursing home residents with dementia	Psychogeriatrics	Germany	Europe	Interaction	Observation /Behavioural analysis	Social interaction coded into four subcategories: (i) verbal interaction; (ii) non-verbal interaction–touch; (iii) non-verbal interaction–line of gaze; and (iv) non-verbal interaction–body posture. Further differentiated whether the behaviour was self-directed or directed towards a person, an animal, or an object	Participant > dog	Observer/ experimenter	DAI/DAI simulation
**Wohlfarth (2014)**	An investigation into the efficacy of therapy dogs on reading performance in 6-7 year old children	Human-Animal Interaction Bulletin	Germany	Europe	Interaction	Observation /Behavioural analysis	Human-dog interaction on a documentation sheet developed for the study. Visual or physical contact to the dog (petting and touching)	Participant > dog	Observer/ experimenter	DAI/DAI simulation
